# Toddlers Using Tablets: They Engage, Play, and Learn

**DOI:** 10.3389/fpsyg.2021.564479

**Published:** 2021-05-31

**Authors:** Mary L. Courage, Lynn M. Frizzell, Colin S. Walsh, Megan Smith

**Affiliations:** Department of Psychology, Memorial University of Newfoundland, St. John’s, NL, Canada

**Keywords:** apps, attention, e-books, executive functions, spatial skill learning, toddlers, touchscreen device

## Abstract

Although very young children have unprecedented access to touchscreen devices, there is limited research on how successfully they operate these devices for play and learning. For infants and toddlers, whose cognitive, fine motor, and executive functions are immature, several basic questions are significant: (1) Can they operate a tablet purposefully to achieve a goal? (2) Can they acquire operating skills and learn new information from commercially available apps? (3) Do individual differences in executive functioning predict success in using and learning from the apps? Accordingly, 31 2-year-olds (*M* = 30.82 month, *SD* = 2.70; 18 female) were compared with 29 3-year-olds (*M* = 40.92 month, *SD* = 4.82; 13 female) using two commercially available apps with different task and skill requirements: (1) a shape matching app performed across 3 days, and (2) a storybook app with performance compared to that on a matched paper storybook. Children also completed (3) the Minnesota Executive Functioning Scale. An adult provided minimal scaffolding throughout. The results showed: (1) toddlers could provide simple goal-directed touch gestures and the manual interactions needed to operate the tablet (2) after controlling for prior experience with shape matching, toddlers’ increased success and efficiency, made fewer errors, decreased completion times, and required less scaffolding across trials, (3) they recognized more story content from the e-book and were less distracted than from the paper book, (4) executive functioning contributed unique variance to the outcome measures on both apps, and (5) 3-year-olds outperformed 2-year-olds on all measures. The results are discussed in terms of the potential of interactive devices to support toddlers’ learning.

## Introduction

The speed with which interactive mobile media technology has evolved and the extent of its adoption into homes and schools has raised enthusiasm and concern among parents and professionals in medicine, science and education (Kucirkova and Zuckerman, 2017; Rideout, 2017; Pila et al., 2019). As these new technologies offer the potential for extraordinary connectivity but also significant distraction and pre-occupation, many of the questions that were raised about the effects of television and video on very young children’s cognitive and social development are being asked again about tablets, smartphones, and gaming devices (see Barr and Linebarger, 2017; Blumberg and Brooks, 2017). One reason for this renewed inquiry is that because of their portability and touch screen capability in particular, these devices are potentially far more intrusive into children’s daily lives than is television. A second reason is that unlike television, touchscreen devices are interactive media and are fairly easy for young children to operate on their own. The devices can easily engage their attention, respond contingently to them, and elicit verbal replies or actions, all of which could support or enhance learning, even in toddlers, when used judiciously with well-designed content (Hirsh-Pasek et al., 2015; Radesky et al., 2015). Some apps also allow for cooperative use that could foster joint attention and engagement with others during play or learning activities (Wholwend, 2015; Lytle et al., 2018; McClure et al., 2018).

A report from *Common Sense Media* confirmed the ubiquity of these devices in the everyday lives of very young children (Rideout, 2017). At that time, an estimated 98% of homes in the United States with children under the age of 8 years had at least one mobile device such as a tablet, smartphone, e-reader, or gaming console. Among children under 2-years of age in the sample, 46% had experience with a touchscreen device. Kabali et al. (2015) reported a 75% usage rate in a sample of children between 12 and 36 months old from low-income, minority group families. More recently, Levine et al. (2019) reported a 60% rate of usage in a sample of children under 36-months of age. Rates reported in several European and Asian samples were similarly high (Ahearne et al., 2015; Cristia and Seidl, 2015; Bedford et al., 2016; Chang et al., 2018; Chindamo et al., 2019). Although the methodology, sample sizes, demographics, and the age ranges varied across these reports, it appears that internationally, more than half of children under 3 years are regular users of interactive media devices. Collectively, these studies also indicated that although traditional television is still their media platform of choice, the amount of time that infant and toddler users spend with interactive devices is increasing and ranges from about 10 to 45 min per day. Parents report in surveys and interviews that children used the devices mainly to access TV and video content through streaming services and YouTube, and to a lesser extent, for interactive e-storybooks, games, and other apps–about 50% of the time when on their own (Nevski and Siibak, 2016; O’Connor and Fotakopoulou, 2016; Rideout, 2017; Levine et al., 2019).

### Mobile Media Technology and the Youngest Users: What Do We Know?

There is evidence that the judicious use of interactive mobile devices can be beneficial to the acquisition of language, literacy, and STEM concepts in older preschool and kindergarten children (e.g., Berkowitz et al., 2015; Bus et al., 2015; Alade et al., 2016; Huber et al., 2016; Herodotou, 2017). These findings have raised the question of whether similar benefits might also be possible for infants and toddlers. Survey data indicate that although most parents have mixed views about giving toddlers access to mobile media, many acknowledge that tablets and other devices are here to stay and that even these very young children need to become familiar with them and to acquire basic user skills. When asked, about half of them also report that well-designed apps can enable new and independent learning, promote creativity, provide entertainment, and can sooth or distract children when distressed (Nevski and Siibak, 2016; O’Connor and Fotakopoulou, 2016; Radesky et al., 2016; Rideout, 2017; Levine et al., 2019). Some parents also see benefit in using video chat apps to maintain communication with absent family and friends (McClure et al., 2015; Chassiakos et al., 2016).

Consistent with these beliefs and expectations, there is experimental evidence that infants and toddlers can learn to imitate simple actions, recognize new words, and solve certain problems (e.g., object retrievals) from interactive video material including video chat (Zack et al., 2013; Roseberry et al., 2014; Choi and Kirkorian, 2016; Kirkorian et al., 2016; Myers et al., 2017; Strouse and Ganea, 2017b). This is most likely to occur when the content directs their attention to important information, increases engagement, and provides them with a sense of achievement. However, there is also evidence from some of these studies that none of this occurs easily or necessarily transfers to new objects or contexts without responsive and contingent support from an adult who scaffolds the toddler during the activity (see Zack and Barr, 2016; Strouse and Ganea, 2017b; Kirkorian, 2018; Troseth et al., 2018). It is important to note that experimental studies are designed to answer particular research questions about learning from interactive media and researchers develop their own testing materials and protocols for this. The control of content and context that this enables makes it preferable to commercially available material, although generalization to real-world apps and viewing conditions is constrained. However, much of children’s exposure to touchscreens at home occurs with commercially available apps, and in varied contexts with or without others. Learning outcomes among children under 3 years using these materials in less controlled conditions is unclear.

Researchers, educators and parents who have expressed concerns about providing touchscreen devices to infants and toddlers point to evidence that children younger than about 3 years have a “transfer deficit.” What this means is that they have difficulty generalizing what they learn in one modality (e.g., from a “live” person or a 3D object) to another modality (e.g., a screen or a 2D representation), although this can be ameliorated by adding contingency, responsiveness, and repetition to the learning context (see Choi et al., 2017; Kirkorian, 2018; Barr, 2019). There are other concerns about potentially harmful associations between toddlers’ use of touchscreens and certain negative developmental outcomes. These include the disruption of stable sleep patterns (Cheung et al., 2017; Chindamo et al., 2019), poor expressive language (van den Heuvel et al., 2019; but see Taylor et al., 2018), and other more general aspects of healthy development assessed by standardized tests (e.g., motor skill, communication, problem solving, personal-social-emotional behavior) (Chassiakos et al., 2016; Madigan et al., 2019). In contrast to most experimental studies, these correlational and survey data are based largely on commercially available content that children use at home under various conditions.

### Toward Resolving Some Basic Questions

The impact of interactive touchscreen use on learning and development in children under 3-years of age is a complex and evolving story (see Reich et al., 2016; Herodotou, 2017). Evidence from research with older preschoolers indicates that only by considering the conjoint effects of the app or video content, the viewing context, and individual child characteristics will fundamental questions be resolved (Barr and Linebarger, 2017; Blumberg and Brooks, 2017; Kucirkova and Zuckerman, 2017). From this broad research agenda, three basic questions suggested by the literature on children younger than 3 years guided the current study. First, there is little information on just how effectively toddlers can operate an interactive touchscreen device. Observations indicate that they are highly attentive to screens and seem able to tap, drag, and swipe to activate simple features even before they have fully developed fine motor control (Aziz et al., 2014; Ahearne et al., 2015; Hourcade et al., 2015; Nacher and Jaen, 2015; Bedford et al., 2016; Samarakoon et al., 2019; Souto et al., 2020). However, random touching and tapping to produce any interesting effect might developmentally precede deliberate, purposeful activation of an app or feature to achieve a specific goal (Adolph and Franchak, 2017). Second, once toddlers can engage purposefully with touchscreen devices, the next questions concern whether, what, and under what conditions they can learn content from them (see Lovato and Waxman, 2016; Kucirkova and Zuckerman, 2017). Although 2-year-olds can acquire new information from traditional screen media when the content is well designed and age-appropriate and when learning is scaffolded (Kirkorian, 2018; Barr, 2019), there is little evidence on additional benefits or detriments from using the newer interactive devices with toddlers (Reich et al., 2016). Third, as the use of interactive devices can tax toddlers limited cognitive resources (Zack et al., 2013; Fisch, 2017; Russo-Johnson et al., 2017), the maturity of their executive functions might predict device success for any particular child. Executive functions are a set of interrelated cognitive processes (inhibition, working memory, cognitive flexibility) that enables self-regulation of thought, feeling, and behavior in a range of activities (Diamond, 2013). As such, they are relevant to understanding how children operate and learn from interactive devices. Those with more mature executive functions might be better able to adapt to the extra cognitive load, keep more information in mind, sustain their attention to a goal, and resist distraction.

### Overview of the Current Study

The study addressed three basic research questions about toddlers use of, and learning from an interactive tablet device: (1) Can they engage with or operate it purposefully to attain a goal? (2) Can they learn operating skills and content from selected commercially available apps? (3) Do individual differences in their executive functions predict success in using, and learning from the apps? To address the first two questions, a group of 2-year-old toddlers was compared with a group of 3-year-olds on their performance using two different, commercially available apps in the supportive presence of an adult. One app primarily drew upon their visuospatial and motor skill in using a tablet to solve a series of shape matching puzzles over three learning opportunities. The second app required less visuospatial and motor skill but drew primarily on their story comprehension and their retention of information from an electronic storybook. The third question on individual differences was addressed with the Minnesota Executive Function Scale (MEFS) (Carlson and Zelazo, 2014), a tablet-based version of the standard Dimensional Change Card Sort Test (DCCS) of executive functioning.

As each app had its own task, skill, and cognitive requirements, the data from each one was expected to make its own contribution to the literature on the efficacy of toddlers’ use of interactive devices. Each app had its own procedure, dependent measures, and plan for analysis as detailed below. The particular shape matching and storybook apps were selected because of the importance of the particular cognitive content that they required. Specifically, in their traditional or concrete formats (e.g., blocks, shape sorters; paper storybooks), there is evidence that both early play and experience with shapes and with shared storybook reading strongly predict spatial and mathematical understanding and emergent literacy, respectively, at school entry and beyond (Mol and Bus, 2011; Verdine et al., 2014a, 2016; Bus et al., 2015; Zosh et al., 2015; Huber et al., 2019; Kucirkova, 2019). As newer digital formats become increasingly available, whether they also provide toddlers with these central foundations for school readiness will become an important question. Moreover, both the shape matching and storybook apps were expected to draw on children’s executive functions such as selective attention, working memory and resistance to distraction, demands that could put them at a performance disadvantage on many e-learning tasks (Verdine et al., 2014b; Fisch, 2017; Russo-Johnson et al., 2017). Although advances in executive functioning are related to age, there are individual differences within age that result from various neurobiological, genetic, and social factors (Johansson et al., 2015; Bell and Cuevas, 2016).

Finally, the focus of the study was on the 2-year-olds, as little is known about the efficiency of touchscreen interactions in children this young. The literature that there is suggests that 24 months is about the earliest age that the beginning of purposeful use might be expected. However, this has not been examined using commercially available educational apps. This gap is significant as many apps in the marketplace target toddlers, often with unsubstantiated claims of their potential for learning (Shuler, 2012; Samarakoon et al., 2019). Toddlers may be especially vulnerable as they have more difficulty in learning from screens than from a “live” or a concrete equivalent source. The 3-year-olds, having largely moved beyond the transfer deficit and with more mature fine motor skills, were included as a comparison group against which to benchmark toddlers’ performance. Although 3-year-olds should perform better that 2-year-olds on these or most tasks, the age range spans an important transition in the development of screen learning.

## Materials and Methods

### Participants

Parents of 2- and 3-year-olds enrolled in local childcare centers were invited to participate in the study and 71 provided consents. Eleven children were excluded because (a) they were unwilling to participate (*n* = 7), (b) technical difficulty during the procedure (*n* = 2), (c) they were outside the target age range (*n* = 1), or (d) had a cognitive impairment (*n* = 1). A total of 60 children (29 boys, 31 girls) comprised the final sample. The mean age was 35.87 months (*SD* = 6.60) and ranged from 26.51 to 46.77 months. Children were divided on a median split (35.37 month) into an older age group (29, 3-year-olds; *M* = 40.92 month, *SD* = 4.82; 13 female) and a younger age group (31, 2-year-olds; *M* = 30.82 month, *SD* = 2.70; 18 female). Most children (*n* = 52, 86.7%) self-reported previous experience with a tablet that they used for “games.” Children were from a well-educated sample of parents, of whom 91% had a university degree. Consistent with the local population from which the sample was drawn, 86.7% of them were White European, with the remaining participants of African (6.7%) and Asian (6.6%) heritage. The children in the final sample were typically developing with no known developmental issues.

### Materials and Measures

The shape matching and storybook apps and the MEFS were presented to the participants on an 9.7-inch Apple 3 iPad tablet. A tablet device was selected because it is in most common use among very young children for play and learning, either when alone or with others (Bedford et al., 2016; Rideout, 2017). Although smartphones and other gaming platforms are interactive and share many tablet features, the tablet’s larger screen interface make it best suited to toddlers’ immature fine motor skill and control (Bedford et al., 2016; Brakke and Pacheco, 2019). The shape matching and storybook apps were selected to examine research questions 1 and 2.

#### Shape Matching Materials

**FIGURE 1:**
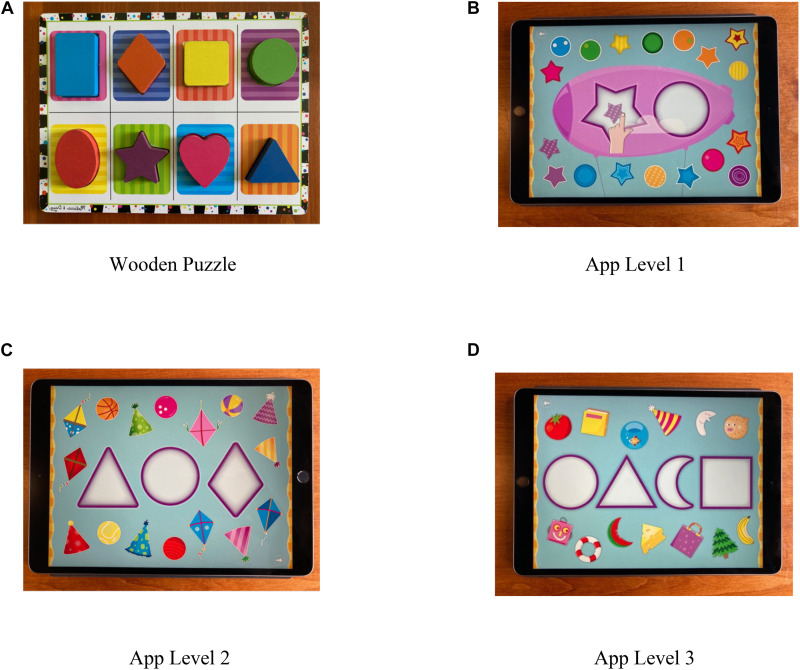
Wooden **(A)** and electronic **(B–D)** shape matching puzzles. The wood puzzle is from the *Melissa and Doug Toy Company*, Wilton, CT, United States. The electronic puzzles are apps from TinyHands, tinyhandsapps.com.

A systematic search of relevant websites was conducted to find an age-appropriate, shape matching app that also appeared to meet the criteria for an educational app–active, engaging, meaningful to the user, and interactive (e.g., Hirsh-Pasek et al., 2015; Dore et al., 2019). The final selection was made from the TinyHands series, targeted to 2- and 3-year-olds (see Figure 1). The task consisted of a number of shapes, each to be dragged from the perimeter of the display and dropped into its corresponding location on the screen. The shape matching puzzles had three levels of difficulty: Level 1 had two shapes with 18 pieces to be placed, Level 2 had 3 shapes with 16 pieces, and Level 3 had 4 shapes with 13 pieces, for a total of 47 pieces. The pieces in Level 1 were canonical whereas those in Levels 2 and 3 were embedded; familiar items shaped approximately like one of the background shapes (e.g., a Christmas tree that fitted in the triangle location) (see Verdine et al., 2016). When a shape was correctly placed it ‘‘faded’’ onto the background and a brief musical trill was heard. Incorrectly placed pieces elicited a ‘‘thump’’ sound and drifted back to their initial location on the screen. Once all the pieces were placed, a cartoon rocket towing balloons flew across the screen. The child could pop the balloons with repeated finger tap gestures. Although available from the App Store (TinyHands)^1^, none of the children reported familiarity with it when asked. In addition to the app, a conceptually similar wooden shape matching puzzle was used as a pre-test to control for each child’s level of prior knowledge of common shapes and their skill in fitting each piece into its corresponding location on a board. As shape matching toys are commonly available, some participants might have had more prior experience with them than others. The wooden shape puzzle was an 8 pieces, commercially available toy (Melissa and Doug^TM^, Inc.), age appropriate for toddlers and preschoolers (see Figure 1). For both the app and wooden versions of the puzzles, although not directly comparable, children were asked to match a common shape (e.g., circle, triangle) with its corresponding location on a background.

#### Storybook Comprehension Materials

**FIGURE 2:**
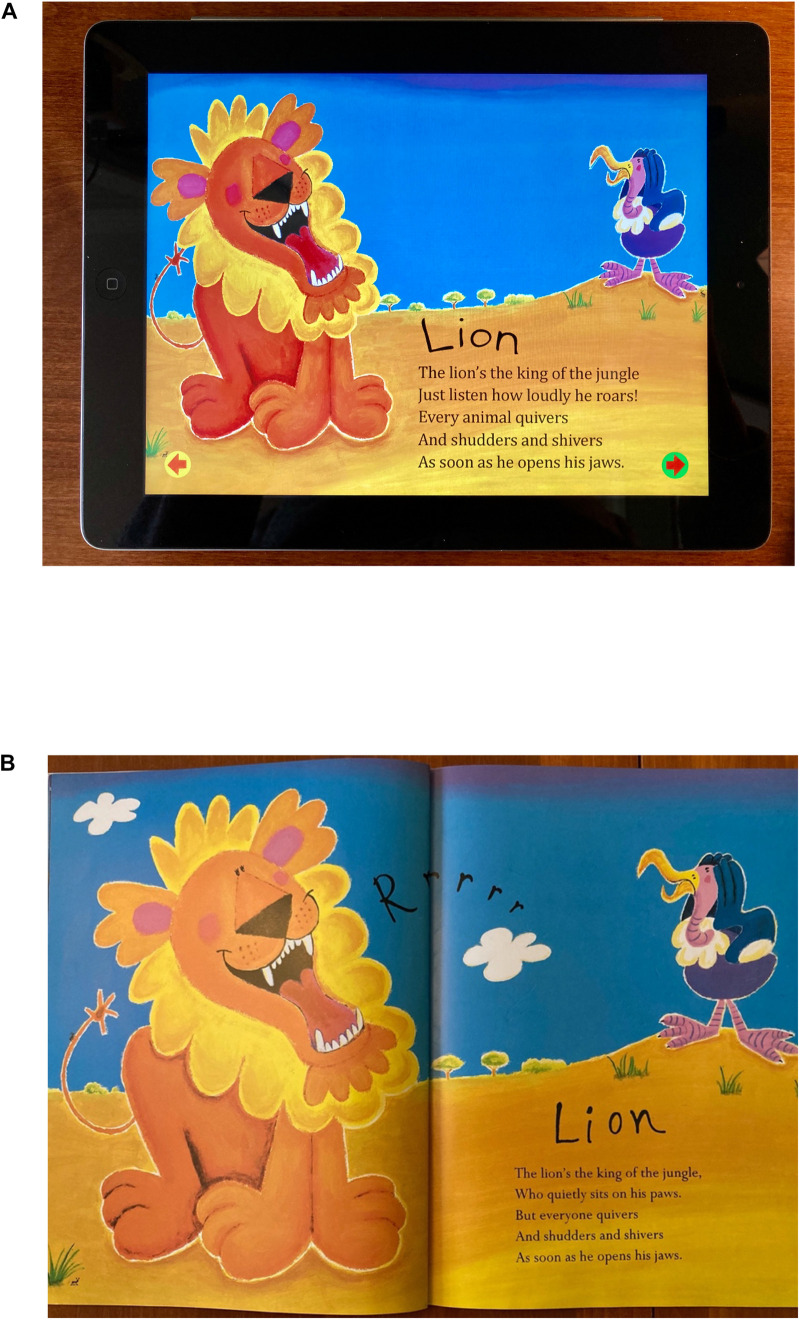
Electronic **(A)** and paper **(B)** illustrations from *Rumble in the Jungle* written by Giles Andreae and illustrated by David Wojtowycz. London, United Kingdom: Orchard Books, 2009.

Similar considerations went into the selection of the storybook app. In addition, the storybook app had to be available in a print format with near identical images and text so that the two formats could be compared directly. A commercially available storybook available in paper and electronic formats was selected: *Rumble in the Jungle* written by Giles Andreae and illustrated by David Wojtowycz. The book provides a humorous description in verse of the activity of familiar jungle animals as they roam about in the night. It is colorfully illustrated and age appropriate for 2- and 3-year-olds. The two book formats were closely matched in length, text, and illustration (see Figure 2). The paper book had 18 pages; the e-book had 12 pages (screens). The two formats presented identical images in composition and number and each story had 377 words of text. The functional page/screen size of the e-book was smaller (8 in × 6 in) than the paper version (11.77 in × 10.5 in) and showed only one page at a time. In addition, the e-book had several multimedia and interactive features per page. Multimedia features included a variety of integrated sounds and animations (background music, highlighted text) that enhanced the narration during reading. The interactive features also provided additional information or options to augment the story (e.g., animal sounds or movements) but also required the child to switch attention away from the narration to activate the feature with a finger tap (see Takacs et al., 2015). None of the interactive features were essential to follow the story narrative and all were consistent with the story content. Children navigated the e-book by tapping an arrow on the screen.

#### The Minnesota Executive Function Scale

**FIGURE 3:**
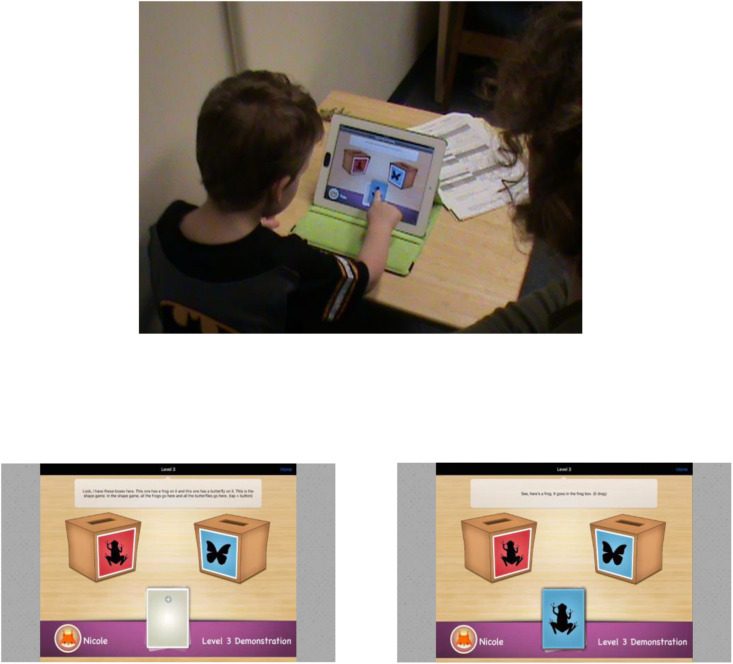
A 3-year-old boy completes a version of the Minnesota Executive Function Scale. Images show the *Minnesota Executive Function Scale* by Carlson and Zelazo (2014), Saint Paul, MN: Reflection Sciences, LLC. Copyright 2014 by Reflection Sciences, LLC. Reprinted with permission.

The MEFS (Reflection Sciences, Inc.) is a tablet-based sorting task modeled on the DCCS (Zelazo, 2006), a widely used test of executive function for preschool children (see Figure 3). Performance on the DCCS (and MEFS) provides an index of the cognitive flexibility component of executive function, but also draws on working memory and inhibition components. In the standard version of the DCCS, children sort a series of bivalent test cards (e.g., blue trucks, red flowers) into boxes, first according to one dimension (e.g., color), and then according to the other (e.g., shape). Most 3-year-olds perseverate during the post-switch phase on the standard task, continuing to sort by the initial dimension. By 5 years, most children switch sorting dimensions when instructed to do so. In the MEFS, bivalent stimuli are presented on a tablet screen and children sort virtual cards into virtual boxes with a finger, first according to one dimension (e.g., color) and then the other (e.g., shape). The task has seven levels of difficulty and takes 4–5 min. If the child cannot drag and drop successfully, but can otherwise indicate the correct box, credit is given for the choice. The reliability and validity of the MEFS for children ages 2–13 years have been reported (Carlson, 2017). The MEFS software algorithm scores and summarizes the data from children’s responses and provides a global measure of executive functioning.

### Procedure

Children were pre-tested on the shape matching wooden puzzle on Day 1 followed by a series of three shape matching app puzzles and the storybook reading. The shape matching puzzles were completed again on Days 2 and 3. Every attempt was made to keep the time between the successive days uniform. However, the time between Days 1 and 2 and between Days 2 and 3 ranged from 1 to 3 days (*M* = 1.52, SD = 0.56; *M* = 1.47, SD = 0.53, respectively), although this did not differ by age group. Children were tested with the MEFS on Day 2 to ensure that they all had practice in using the tablet. The study took place at the childcare centers in a quiet room with a child-sized table and chairs. They were tested individually with a researcher seated beside them to explain and guide the procedure. The puzzle tasks and storybook reading were video recorded for later off-line coding. The study protocol was approved by the institutional research ethics committee.

#### Day 1

After establishing rapport with the child, the researcher explained that they were going to do some puzzles. First, the child was asked to complete the wooden shape matching puzzle. The researcher introduced the task and provided an example (e.g., “The heart piece goes in the heart-shaped spot”) and placed the shape. The child was asked to place the remaining seven pieces. Next, the researcher opened the iPad and asked if he or she had ever used a tablet device and for what purpose. At each of the three levels of the shape matching app, the researcher pointed out how many different shapes there were (i.e., two, three, or four, respectively) and provided an example of dragging a shape to its correct location. A “ghost hand” also dragged and dropped a sample “ghost” shape into each location. The child was then asked to place the shapes. They were praised for correct placements and prompted if they were having difficulty (e.g., repeatedly placing the same piece in an incorrect location). Prompting was kept to a minimum and consisted of pointing out the correspondence between the shape and its location, instructing how to drag and drop the shapes, or refocusing the child’s attention. Every attempt was made to keep such scaffolding standardized and to use similar wording and tone across children. However, there was of necessity some variation in this in order to adapt to the particular child and situation. The researcher then moved on to Levels 2 and 3 of the shape matching app and followed the same procedure.

The next activity was to read the child a story from either the paper or the electronic storybook. The book format was assigned alternately as children became available for testing with the caveat that there be approximately an equal number of each format in each age and gender group. At the beginning, children were asked whether they had heard the *Rumble in the Jungle* story before but none had. Those assigned to the e-storybook group were then instructed how to use the tablet touch screen and to turn the pages. The adult demonstrated the interactive features and encouraged the child to activate them and to look for others during the reading. The story was presented using the “Read to Me” option that was narrated by software voices but allowed the child to view and turn the pages at will. If the child did not turn the page after about 15 s and did not appear to be engaged, the researcher prompted “Ok, let’s turn the page now.” If the child was engaged with the features, he or she was allowed to continue for up to 2 min at which time the child was prompted to move on (“Let’s see what’s next”). The paper book reading followed a similar procedure. The researcher read the story at the same pace as the e-book narration and allowed the child to examine the story illustrations and to turn pages. As with the shape app, scaffolding was kept to a minimum and standardized in wording and tone and but varied somewhat to adapt to the child and the situation. No dialogic prompts were provided. Immediately after the readings, the child was asked five recognition questions about the story. A series of illustrations from the paper book were photographed and laminated as 4 × 6-inch cards. Each child was shown a subset of 4 cards and asked to identify which card matched a question such as “Which of these animals swings from the trees?” The wording of the questions came directly from the storybook text. The entire testing session took about 35 min.

#### Day 2

The researcher repeated the shape matching app instructions and proceeded to guide the child through the three levels as on Day 1. Following this, the researcher administered the MEFS using the standardized protocol (Carlson and Zelazo, 2014). Children began at an age-appropriate level assigned by the MEFS software according to the age information provided, and proceeded to harder or easier levels depending on performance. As seen in Figure 3, the screen displayed two boxes showing two different animals with different colored backgrounds. The researcher turned over a card in the middle of the touch screen that matched the boxes by color or by shape and demonstrated the appropriate sorting rule (by color or by animal). The child was given two practice trials with feedback. In Part A, children sorted by the rule used in the practice trials (e.g., shape). If the child correctly sorted 4 of the 5 cards, he or she moved on to Part B and was instructed to sort by the other dimension (e.g., color). If the child didn’t pass Part A, or failed to sort correctly on Part B, they moved down one level and continued to move down until they passed a level. If the child passed Part B, he or she moved up to the next level, and continued until a failure. Final scores were calculated using the MEFS software algorithm based on accuracy and reaction times (range = 0–100). The Day 2 procedure took about 30 min.

#### Day 3

On day 3, the child once again completed three levels of the shape matching puzzle app. The Day 3 procedure took about 10 min.

### Data Coding

The first two research questions were addressed using tasks presented in app format on a tablet device. Each of the activities had its own dependent measures that were used to infer children’s ability to operate the device purposefully to attain a goal and to learn skills and/or content from the app. These were coded for the analyses from the video recordings as follows:

#### Gestures and Hand Use

In the digital context, a gesture is any physical movement that can be detected and responded to without the use of a pointing device such as a mouse. When the movement is a touch or a tap, it is referred to as a touch gesture (Saffer, 2009; Villamore et al., 2010). In the current study, the following Touch Gestures adapted from Aziz et al. (2014) were coded from the shape matching and the storybook app data as either present or absent: tap, drag/slide, drag and drop, flick/swipe, double or repeated tap, long press, hit. The data were also coded for children’s Manual Interactions with the screen, or the manner in which they used fingers or hands to engage with the target object (e.g., single or multiple fingers). These manual interactions were used to provide a Navigation Skill score, a global measure that reflected the child’s control over the app as evident in the strategy used to move the target from its starting position on the screen to the goal. Navigation Skill was coded as (1) low, (2) moderate, or (3) high. A description of these three coding categories can be seen in Table 1.

**TABLE 1:** Coding scheme for children’s touch gestures, manual interactions, and navigational skill when using the shape matching and storybook apps.

Coded Behavior	Description
Active search/look	Scan screen, focus on a target, shift focus to goal
**^1^Touch Gestures:**	
Tap	Quick up and down motion with a finger; lightly strikes screen
Drag or slide	Place finger on target and move in the desired direction without lifting the finger from the screen
Drag-and-drop	Place finger on target and move in the desired direction without lifting finger from screen then drop target at the goal
Repeat or double tap	Several quick taps in succession on screen
Long press	Touch and hold the target with finger motionless until an action occurs
Swipe or flick	Place finger(s) on screen and quickly flick target in the desired direction
Hit	Quick single slap on the screen with the hand
**^2^Manual Interactions:**	
Full or both hands	Uses full or both hands or palm; hit or slap screen to move or activate target
Multiple fingers, thumb	Uses multiple fingers or thumb to move target; grasp as if to pick up target
Single finger	Uses forefinger to move target
**^3^Navigation Skill:**	
Low (1)	Mixed use of the full hand, palm or heel of the hand, multiple fingers, or thumb to move target to goal; frequent drops
Moderate (2)	Mixed use of single and multiple fingers or thumb to move target to goal; less frequent drops
High (3)	Almost exclusive use of forefinger to move target to goal; infrequent drops

*^1^Touch Gesture: an action of the fingers or hand directed to a target object on the screen. ^2^Manual Interaction: how the child uses the fingers or hands to engage with the target. ^3^Navigation: the strategy used to get the target from its starting position to the goal.*

#### Shape Matching Puzzle Measures

The primary dependent measures of interest for goal attainment on both wooden and app versions were (1) the number of successful placements of the shapes on the first try, (2) the number of concept and (3) motor skill errors made in placing the shapes, (4) the time to complete each puzzle, (5) puzzle efficiency–the ratio of successes to the completion time, (6) the number of prompts the child received during the puzzle completion, and (7) for the app puzzle only, skill in navigating the pieces around the screen coded from 1 (low) to 3 (high). As the wooden shape puzzle was used as a covariate to control for prior user experience, it was completed only on Day 1. The shape matching puzzle app had three levels of difficulty and these were completed on Days 1, 2, and 3, for a total of nine puzzles with 141 pieces (47 per day) to be placed. For the judged measures 25% of the videos selected at random within each age group and were coded by an independent researcher to establish the reliability of the observations using intra-class correlations. These were calculated for numbers of successes, concept and motor errors, prompts, and for skill in navigating the screen. Intra-class correlations ranged from acceptable to excellent: 0.84 for success, 95% CIs (0.52–0.94); 0.98 for concept errors, 95% CIs (0.96–0.99); 0.97 for motor skill errors, CIs (0.95–0.99); 0.98 for prompts, 95% CIs (0.95–0.99); 0.75 for navigation skill 95% CIs (0.30–0.91). For the analyses, the three app puzzles scores were collapsed across difficulty level each day, yielding a total score for Days 1, 2, and 3 as follows:

##### Puzzle success

For the wooden puzzle and for all three levels of the app puzzle, a response was coded as a success only if the child placed a shape in its corresponding correct location on the first try. That is, the child had the conceptual understanding that a particular shape matched a particular location, could see the correspondence between the two, and had the motor or manual skill to place it without difficulty.

##### Puzzle errors

Errors in shape placement on both puzzle tasks were coded as either a concept error or as a motor skill error. A *concept error* occurred when the child attempted to fit a particular piece into an incorrect location, indicating that he or she did not appear to see (or forgot) the correspondence between the shape and its location. A *motor skill error* was coded for the wooden puzzle when the child placed a shape at the correct location, but could not orient it to fit. This indicated that the child knew the shape-location correspondence but lacked the motor skill to place it precisely. If the child had to manipulate the shape very slightly to fit it, this was not coded as an error, whereas separate attempts to place the piece incorrectly (i.e., raised it off the wooden background), in either the same or different locations, were coded as separate motor errors. For the app puzzle, a motor skill error occurred whenever the child began to drag the piece forward along an irregular trajectory or dropped it while dragging it to its location, or attempted to “fling” rather than drag it. These were all coded as separate motor skill errors.

##### Puzzle prompts

Once the child tried to place a puzzle piece in either the wooden or electronic puzzle, but was making repeated errors or beginning to show reluctance or frustration, the researcher provided a verbal prompt to keep the session moving along and to motivate the child (e.g., “Let’s try again,” or “Just move the shape gently with one finger” or “You’re doing really well”) or refocusing his or her attention, (e.g., “Let’s try another shape” or “Where does this one go”). Prompts could also include pointing out the correspondence between the puzzle piece and its location (e.g., “What shape is a Christmas tree?” or “Is the soccer ball shape like a circle or a square?”). Only instructive prompts were included in the analyses. An instructive prompt could vary in length, but was directed to assist with the placement of one particular piece.

##### Puzzle time and efficiency over trials

The *time* to complete the puzzle was recorded from when the child first tried to place a shape to when the last piece of the particular puzzle was placed in its location. Puzzle *efficiency* was a ratio of the successful puzzle placements to the time it took to complete the puzzle. For the shape matching app, the assumption was that across trials an efficient child would place more pieces successfully and do so in less time.

#### Storybook Comprehension Measures

The primary dependent measure was the number of correct recognition questions that the children answered about the contents of the paper and e-storybooks. Either a verbal answer or a point to the correct picture was coded as correct. Other measures of interest were the number of distractions during each reading and the time taken to complete each book reading. A distraction was coded when the child looked off-task away from the book or made a comment that was not related to the story (e.g., “I hear them playing”). A “look” was counted each time the child’s gaze was directed off task, regardless of its length (Lauricella et al., 2015). As with the shape matching puzzle, 25% of the videos were scored for the frequency of off-task distractions, intra-class correlation was 0.96 for looks off-task, 95% CIs (0.94–0.97). Looks toward the adult were not considered a distraction as such social referencing is a typical part of a storybook reading interaction with young children (Richter and Courage, 2017). The time to complete the story was measured from the reading of the title page to the end of the last page.

#### Executive Functioning (MEFS)

All coding was done by the software provided with the testing materials and license. Measures provided were the Baseline level–selected by the program based on the child’s age information; the Highest Level Passed–the last level where the child passed both parts A and B of the test; Ceiling Level–the highest level where the child fails either Part A or B; national norms in standard scores and percentiles, and an RT-Adjusted scores (0–100) based on an algorithm that takes both response accuracy and reaction time into account. Only the RT-Adjusted scores (i.e., MEFS total scores) were used in the analyses here.

## Results

Consistent with the inclusion of two different app activities–shape matching and an e-storybook–the two data sets were independently analyzed and are reported sequentially below.

### Gestures and Hand Use

Examination of the video data revealed that, with adult support 100% of the children were able to actively search the screen array and produce the basic touch gestures (search/look, tap, slide, drag-and-drop) needed to engage with and solve the shape matching app puzzles. However, non-essential gestures, swipe/flick, hit, repeated tap, grasp, were also observed in 58.06, 19.35, 9.67, and 3.22%, respectively, of the toddlers. These non-essential gestures were rare in the older children; 20.69% showed swipe and 0% for hit, repeated tap or grasp. Observation of toddlers’ manual interactions showed that 70.97% used multiple fingers and 38.72% used their full hand to move the shapes. Among 3-year-olds, 24.33% used multiple fingers and none used the full hand. Consistent with this was children’s improvement in navigation skill rated from (1) low, to (2) moderate, to (3) high over the 3 days. A non-parametric Friedman Test of differences among repeated measures was conducted for each age group and the results yielded significant Chi-square values for both groups χ^2^(2) = 27.11, *p* < 0.001; χ^2^(2) = 14.00, *p* = 0.001, respectively. Wilcoxon Signed Rank follow up tests within each age group indicated that 2-year-olds’ navigation skill increased incrementally over the three trials; from Days 1 to 2 and from Day 2 to 3: *Z*s(31) = −3.00, *p* = 0.003 and −3.32, *p* < 0.001. The 3-year-olds improvement was significant between Day 1 and 2: *Z*s(29) = −2.828, *p* = 0.005 but stabilized after that: *Z*s(29) = −1.342, *p* = 0.180.

### Shape Matching Activities

#### Wooden Puzzle

Both age groups performed well on the wooden puzzle pre-test with the 3-year-olds making 94.43% correct placements on Day 1 and the 2-year-olds with 85.43% correct placements (see Table 2). A series of independent sample *t*-tests showed that the older children had significantly more successes out of the 7 pieces than did the younger children, *t*(58) = 2.50, *p* = 0.015, *d* = 0.62. They made significantly fewer concept *t*(58) = 2.92, *p* = 0.005, *d* = 0.71 and motor skill *t*(58) = 2.40, *p* = 0.002, *d* = 0.78 errors, required fewer prompts *t*(58) = 3.85, *p* = 0.001, *d* = 0.90, had faster completion times *t*(58) = 4.27, *p* < 0.001, *d* = 0.95, and greater efficiency scores *t*(57) = 4.43, *p* < 0.001, *d* = 0.91 than did the younger children.

**TABLE 2:** Means and standard deviations of children’s performance on the wooden puzzle pre-test by age and variable.

		Success	Concept Errors	Motor Errors	Prompts	Time (sec)
**Age Measure**						
2 years	M	5.98	1.35	1.29	2.19	59.61
	SD	1.21	1.84	1.34	2.98	29.74
3 years	M	6.61	0.41	0.42	0.59	34.66
	SD	0.66	0.68	0.62	0.98	10.74

*Maximum score = 7 as one piece was used to demonstrate.*

#### App Puzzle

**FIGURE 4:**
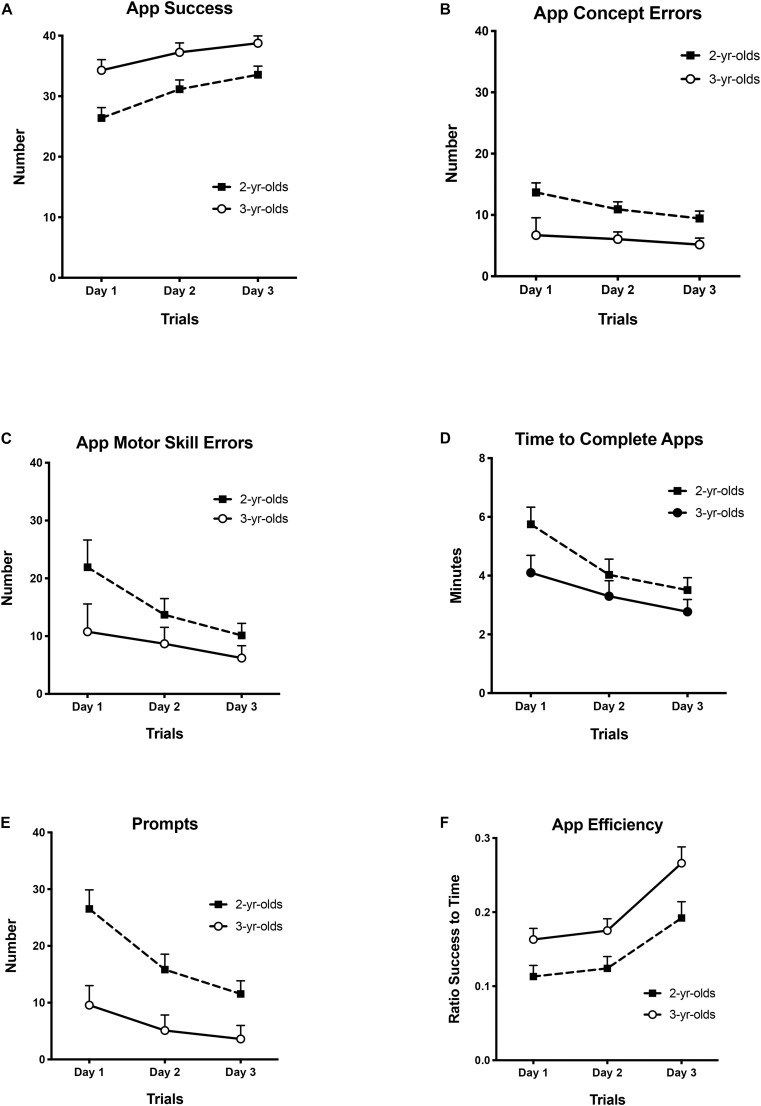
Adjusted means and standard errors for shape matching app performance by age and trials: Number of **(A)** successes (max = 47), **(B)** concept errors, **(C)** motor skill errors, **(D)** completion time in minutes, **(E)** number of prompts, and **(F)** efficiency ratio.

A series of 2 × 3 [Age Group (2 and 3 years) × Trials (Days 1, 2, 3)] repeated measures analyses of co-variance (ANCOVA) were conducted on the six primary dependent variables of interest. As with the wooden puzzle, these were: successful placement, concept errors, motor skill errors, completion times, puzzle efficiency, and the number of prompts provided by the adult. Age group (2 and 3 years) was a between-subjects variable and trials (Days 1, 2, 3) was within subject. To control for children’s previous experience with shape puzzle toys at home, their pre-test performance on the comparable baseline wood puzzle measure was the covariate in each of the ANCOVAs. Where outcome variable distributions were skewed (concept errors, motor errors, completion times, adult prompts), normalization was achieved with log10 transformations of the data. Bonferroni corrections were used to adjust for the number of comparisons in all of the analyses. The effect of gender was assessed with an ANCOVA for the six measures of interest and was not significant in any case (*p*-values for the models ranged from 0.211 to 0.901). Therefore, the data were collapsed across gender in the main analyses. The adjusted means and standard errors for the six outcome measures on the shape app over trials are shown in Figure 4.

##### App success

The dependent measure was the total number of shapes out of a maximum of 47 that children placed correctly on the first try on each of the three successive days. The results of the ANCOVA showed that the covariate was not significant: *F*(1, 56) = 2.39, *p* = 0.127. There was a significant main effect of age *F*(1, 56) = 8.84, *p* = 0.004, η^2^ = 0.136, that reflected the fact that the older group had more successful placements than did the younger group There was also a significant main effect of trials *F*(2,112) = 4.79, *p* = 0.010, η^2^ = 0.079. *Post hoc* pair-wise comparisons indicated that children’s performance improved significantly across trials; there were more successes on Day 3 than on Day 2 (*p* = 0.001) and more on Day 2 than on Day 1 (*p* < 0.001). Successes on Days 3 and 1 also differed significantly (*p* < 0.001) (see Figure 4A).

##### App concept errors

The dependent measure was the total number of first incorrect shape placements made on each day. The ANCOVA showed that the covariate was not significant *F*(1, 56) = 0.962, *p* = 0.333. There were significant main effects of age *F*(1, 56) = 9.14, *p* = 0.004, η^2^ = 0.191 and trials *F*(2, 112) = 8.59, *p* < 0.001, η^2^ = 0.177. *Post hoc* pairwise comparisons indicated that although, older children made significantly fewer concept errors than did younger children, both age groups decreased the number of errors they made on the app puzzle from Days 1 to 2 (*p* = 0.007) and from Days 1 to 3 (*p* = 0.001) but showed no further decrease from Days 2 to 3 (*p* = 0.072) (see Figure 4B).

##### App motor skill errors

The dependent measure was the number of incorrect shape placements made because of a motor skill error on each of the three successive days. The ANCOVA showed that the covariate was significant: *F*(1, 55) = 6.22, *p* ≤ 0.001,η^2^ = 0.102, meaning that prior experience reflected in the wooden puzzle pre-test had a significant effect on app motor errors. There was also a significant main effect of trials *F*(2, 110) = 20.37, *p* < 0.001, η^2^ = 0.270, showing that in general, children made fewer errors across trials. This main effect was qualified by a significant Trials × Age interaction *F*(2, 110) = 3.55, *p* = 0.032, η^2^ = 0.061. *Post hoc* independent sample *t* tests indicated that although the older children made significantly fewer motor errors than did the younger children on both Day 1, *t*(57) = 2.20, *p* = 0.032; *d* = 0.55; and on Day 2, *t*(57) = 2.29, *p* = 0.025, *d* = 0.58, by Day 3, there was no significant difference between the two ages *t*(57) = 1.77, *p* = 0.084 (see Figure 4C).

##### App times

The dependent measure was the total amount of time that children took to complete the app puzzles on each of the 3 days. The results of the ANCOVA showed that the covariate was significant: *F*(1, 56) = 19.86, *p* ≤ 0.001, η^2^ = 0.262, meaning that prior experience with the wooden puzzle pre-test had a significant effect on app completion time. There was a significant main effect of age *F*(1, 56) = 4.33, *p* = 0.042, η^2^ = 0.072, as older children took significantly less time to complete the app puzzles than did the younger children. There was no significant main effect or interaction involving trials *F*(2, 112) = 1.61, *p* = 0.204; *F*(2, 112) = 1.03, *p* = 0.360, respectively. However, examination of the adjusted means indicated a systematic decrease in time to complete the app across the three trials in both age groups (2 year-olds: *M*s = 5.75 min, 4.03 min, and 3.51 min, respectively; 3-year-olds: *M*s = 4.10 min, 3.30 min, and 2.77 min, respectively). Consistent with this, if the planned pairwise comparisons are considered, they further supported the prediction that completion times decreased markedly across days; longer on Day 1 than on Day 2 (*p* < 0.001), and longer on Day 2 than Day 3 (*p* = 0.001). Days 1 and 3 also differed significantly (*p* ≤ 0.001). See Figure 4D.

##### Prompts during app use

The dependent measure was the total number of prompts provided by the researcher during the app puzzles on each day. The ANCOVA showed that the covariate was not significant *F*(1, 56) = 3.10, *p* = 0.084. There were significant main effects of age *F*(1, 56) = 10.19, *p* = 0.002, η^2^ = 0.154 and trials *F*(2, 112) = 16.19, *p* < 0.001, η^2^ = 0.224. *Post hoc* pairwise comparisons indicated that although older children needed significantly fewer prompts than did younger children, both age groups were given significantly fewer prompts across successive days, Days 1 to 2 (*p* < 0.001) and Days 2 to 3 (*p* < 0.001). Day 1 also differed significantly from Day 3 (*p* < 0.001) (see Figure 4E).

##### App efficiency

The measure was a ratio of the successful placements to the time taken to complete the puzzles on each of the three successive days. The ANCOVA showed that the covariate was significant: *F*(1, 56) = 13.60, *p* = 0.001. There were also significant main effects of age *F*(1, 56) = 5.58, *p* = 0.022, η^2^ = 0.091, and trials *F*(2, 112) = 7.24, *p* = 0.001, η^2^ = 0.114. *Post hoc* pairwise comparisons showed that apart from the older children being significantly more efficient on the app puzzle than were the younger children, both ages became increasingly efficient across trials from Days 1 to 2 (*p* < 0.001) and from Days 2 to 3 (*p* < 0.001) Days 1 and 3 also differed significantly (*p* < 0.001) (see Figure 4F).

In sum, both age groups of children showed goal-directed performance with the shape matching app puzzles and improved their performance over the three days of testing. Older children generally performed better than the younger children; they had higher success scores, made fewer conceptual and motor skill errors, were faster and more efficient using the app, and required fewer prompts from the adult. Moreover, on all 3 days, there was significant negative Pearson *r* correlations between app success scores and both conceptual and motor skill error scores, *r*s(59) ranged from −0.552 to −0.763, all *p* < 0.001.

### Storybook Comprehension

**FIGURE 5:**
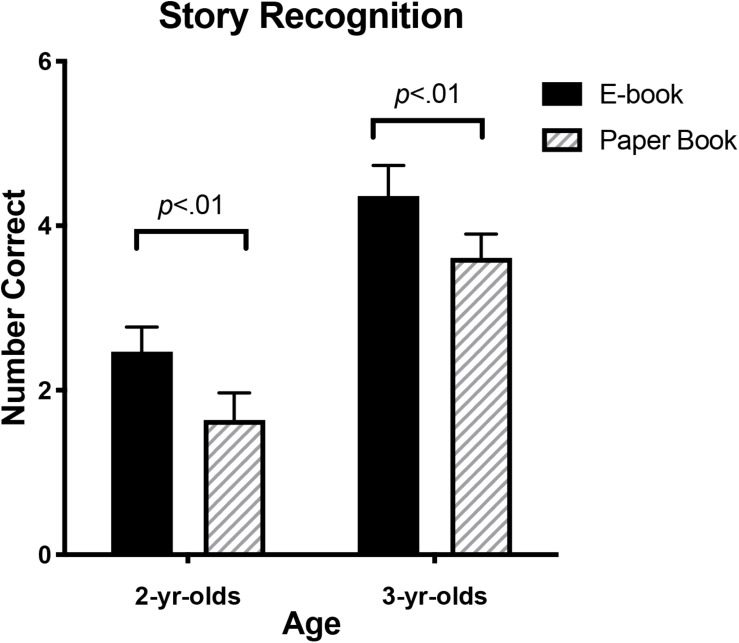
Means and standard errors for children’s recognition memory scores by age and book format.

The e-book app made little demand on children’s motor skill beyond simple active looking or search and tap gestures. All children were able to do this to turn the pages and to seek out and activate the interactive features. To evaluate children’s retention of story content, a 2 × 3 [Age Group (2 and 3 years) × Book Format (electronic, paper bound)] analysis of variance (ANOVA) was conducted on the recognition of story information data. The results showed a significant main effect of age *F*(1, 56) = 32.74, *p* < 0.001, η^2^ = 0.369, indicating that the 3-year-olds had higher recognition scores than did the 2-year-olds. There was also a significant main effect of book format *F*(1, 56) = 7.44, *p* = 0.008, η^2^ = 0.117. Children recognized more information from the e-book than the paper book. The means and standard deviations for each age and book format are shown in Figure 5 and reflect the fact that the 2-year-olds recognized more of the story content items following the e-book (*M* = 2.65, SD = 1.27) than the paper book (*M* = 1.64, SD = 1.00), as did the 3-year-olds (*M* = 4.36, SD = 1.03; *M* = 3.61, SD = 1.41), respectively.

The e-book took significantly longer to complete than did the paper book for both age groups. For the 2-year-olds: e-book *M* = 7.39 min, SD = 1.72; paper book *M* = 5.55 min, SD = 1.67; *t*(28) = 3.31, *p* = 0.003, *d* = 0.71. For the 3-year-olds: e-book *M* = 5.97 min, SD = 0.71; paper book *M* = 5.25 min, SD = 0.98; *t*(27) = 2.10, *p* = 0.046, *d* = 0.48. Moreover, the children were more attentive to the e-book than to the paper book, as reflected in the lower number of distractions (i.e., looks away from the book or off-task comments) they showed during the readings. These data were analyzed in a 2 × 2 (Age Group [2 and 3 years] × Book Format [electronic, paper] analysis of variance (ANOVA). The results indicated only a significant main effect of book format *F*(1, 57) = 6.44, *p* = 0.014, η^2^ = 0.113. Children at both ages in the paper book condition were more distracted off task during the readings than were those in the e-book condition e-book *M* = 4.89, SD = 5.63; paper book *M* = 8.90, SD = 7.59. Consistent with this, children were highly engaged in using the interactive features of the e-book, although a direct comparison with the paper book was not possible. Overall, they frequently activated the features (*M* = 27.89, SD = 16.23 times; range from 7 to 78) during the e-book reading. There was no age difference (2 year: *M* = 28.82, SD = 14.24; 3 year: *M* = 26.30, SD = 19.90) in the number of activations *t*(25) = 0.384, *p* = 0.704.

### Executive Functions

The role of children’s executive functioning in their successful performance on the two tablet-based tasks was assessed with separate multiple regression analyses of those data. The outcome measure of executive functioning was the RT-Adjusted score as calculated from the MEFS software. Preliminary evaluation of the assumptions underlying the use of regression (adequate sample size, normality of the outcome variable distribution, multi-co-linearity, outliers, and linearity) were found to have been met.

#### Shape Matching App

To examine the role of children’s executive functioning and age on their total puzzle success score summed across the 3 days on the shape matching app, a hierarchical linear regression was conducted. Children’s age in months and their MEFS total scores were the predictors in the analysis of their cumulative puzzle success summed across the 3 days. Success on the wooden puzzle pre-test was the control variable and was entered into Block 1 of the regression. This model was significant *F*(1,51) = 5.48, *p* = 0.023 and explained 9.7% of the variance in the app puzzle success data. With age in months and executive functioning entered as predictors in Block 2, the total variance explained by the model as a whole increased to 47.2%. The inclusion of those predictor variables explained an additional 37.5% of the variance in cumulative success after controlling for puzzle pre-test success, *R*^2^ Change = 0.375, *F*(2,49) = 17.37; *p* < 0.001. In the final adjusted model, MEFS made a unique and significant contribution, *t*(49) = 3.54, *p* < 0.001, β = 0.488 whereas age in months did not, *t*(49) = 1.27, *p* = 0.205, β = 0.180 (see Table 3). In sum, with puzzle pre-test success controlled, results of the regression indicated that the maturity of children’s executive functions was a better predictors of puzzle app success than was age alone.

**TABLE 3:** Hierarchical regression analysis for the variables predicting successful shape matching app placements as entered into Model 1 and Model 2.

Model	Variable	B	SE B	B eta	*T*	*p*
1	Wood Puzzle success	5.75	2.46	0.31	2.34	0.023*
2	Age (months)	0.67	0.53	0.18	1.29	0.203
	Wood puzzle success	3.87	1.96	0.21	1.97	0.054
	Total MEFS	0.828	0.23	0.49	3.54	0.001**

p < 0.05*. p < 0.01**.

#### Storybook App

To examine the role of children’s executive functioning, book format, and age in months on their total story recognition scores, a hierarchical linear regression was conducted. A book format × executive functioning interaction term was also computed. Children’s age in months, their book format assignment, and their MEFS total scores were the predictors of recognition and entered into Block 1 of the regression. The model was significant *F*(3,52) = 14.99, *p* < 0.001 and explained 47.9% (*R*^2^ = 47.9%) of the variance in the recognition data. The addition of a book format × executive functioning (MEFS) interaction into Block 2 did not add significantly to the total variance explained by the model as a whole, *R*^2^ Change = 0.001, *F*(1,48) = 0.052; *p* = 0.821. In the final adjusted model, age made the largest unique and significant contribution, *t*(49) = 2.65, *p* = 0.011, β = 0.375. Total MEFS score also made a unique, significant contribution to the model *t*(49) = 2.58, *p* = 0.013, β = 0.360, as did book format *t*(49) = 2.32, *p* = 0.024, β = 0.248 (see Table 4). In sum, children’s increasing age and executive function maturity and their storybook assignment to the e-book condition all contributing to successful recognition.

**TABLE 4:** Hierarchical regression analysis for the variables predicting recognition of the storybook app content as entered into Model 1 and Model 2.

Model	Variable	B	SE B	B eta	*t*	*p*
1	Book format	0.77	0.33	0.25	2.32	0.024*
	Age (months)	0.09	0.03	0.38	2.65	0.011*
	MEFS	0.04	0.02	0.36	2.58	0.013*
2	Book format	0.75	0.33	0.25	2.29	0.026*
	Age (months)	0.09	0.04	0.37	2.57	0.013*
	MEFS	0.04	0.02	0.39	2.14	0.037*
	Book × MEFS	–0.01	0.02	–0.03	–0.24	0.821

p < 0.05*.

## Discussion

Consistent with data from many countries around the world, almost 90% of the 2- and 3-year-olds in this study reported having previously used a tablet device. As the software developed for these devices is designed to be compelling, engaging, and can be matched to the skills and interest of the individual child, they have the potential to support and even enhance learning when used with appropriate content and adult oversight. Given the many empirical questions that remain to be answered about this possibility, the purpose of the current study was to address three basic questions: First, can toddlers operate a touchscreen device purposefully to attain a goal? Second, can they acquire operating skills and learn content from age-appropriate apps? Third, do individual differences in their executive functions predict success in using, and learning from the apps? Accordingly, a group of 2-year-old toddlers was compared with a group of 3-year-olds as they used an Apple iPad 3 to interact with two commercially available apps with different task and cognitive requirements–a shape matching puzzle and an e-storybook. Although the limited literature on how toddlers actually use tablet devices made the study exploratory, the expectation based on their attention to, and learning from traditional television and video content was that in the supportive presence of an adult, they would engage with both of the apps purposefully and acquire new skills and content, but that the maturity of their executive functioning would predict their success. The 3-year-olds were included as a standard against which to compare the toddlers’ performance. As children over 3 years have largely resolved the transfer deficit, have more mature executive and cognitive functions and better fine motor control, they were expected to outperform the toddlers on all measures.

Concerning the first research question, most of the toddlers were able to use both of the apps purposefully, although their performance was below ceiling on both, with 70.64% successful placements out of 47 shapes on Day 3 and 53.0% correct recognitions out of five questions on the e-storybook. All had the basic search/look, tap, drag/slide, and drag-and-drop gestures in their repertoire and used them on at least some of the nine shape matching puzzles and on the e-storybook. Of these, drag-and-drop was the most challenging and accounted for numerous motor skill errors on the shape matching app as children tried to navigate the pieces to the intended location. The most effective strategy was to use a forefinger to lightly drag the shape directly toward the corresponding location and then drop it. Less effective strategies included using a thumb, several fingers, or the whole hand to move the piece. Others were pressing too firmly on the shape to be moved which slowed it down, tapping the piece repeatedly, trying to grasp it as if to pick it up, or using a forefinger to initiate movement but then “swiping” or “flinging” the piece toward its location. Most older children adopted the forefinger strategy soon after being instructed to do so. Their most frequent error was to drop a shape before reaching the target location, causing it to drift back to its starting point. The younger children more often used the less effective gestures and manual interactions even when instructed otherwise.

Comparable gestural, manual, and navigational skills have been reported in the few other studies that included toddlers (Aziz et al., 2014; Ahearne et al., 2015; Hourcade et al., 2015; Nacher and Jaen, 2015; Samarakoon et al., 2019). Some included the more difficult gestures for toddlers to execute such as drag-and-drop, but also free rotate and pinch and stretch to resize. Typically, 2- and even some 3-year-olds struggled with these with only about 30–60% achieving success. As the shape matching and e-book apps in the current study were selected to be both age appropriate and educational, they did not require these more difficult gestures beyond drag-and-drop. An important point to be taken from this is that when designing apps for toddlers, it is critical to incorporate only those gestures that they can readily produce to control the app and master the task. One contribution of the results reported here is the identification of a suite of touch gestures that can be used in any app to enhance learning, without including more difficult gestures that might drain toddlers’ cognitive resources and diminish task performance.

Concerning the second research question, even the youngest children showed more precise manual interactions with practice, increasing their reliance on single-finger use with less use of the full hand and/or multiple fingers. This was apparent in their improved navigational skill in moving the pieces about the screen over the 3 days. Coincident with this, toddlers improved their shape matching outcomes across the three learning opportunities, even when their prior experience with shape puzzles was controlled. They increased their successful first placements by 15.89% and decreased the number of first concept and motor skill errors by 41.27 and 53.94%, respectively. The reduction in concept errors was notable as it indicated that children improved their shape identification and visuospatial matching along with motor and navigation skill over the same period. They also took 39.68% less time to complete the shape matching puzzles and received 56.49% fewer prompts. Their growing proficiency was also evident in their increased success/time ratios by 41.56%; making more successful placements in less time. These abilities are generally consistent with observational and survey data in which toddlers’ have been rated as showing at least a “moderate” level of skill on the basic touch gestures, that with advances in fine motor control and experience, became better articulated and deployed (Aziz et al., 2014; Ahearne et al., 2015; Cristia and Seidl, 2015; Hourcade et al., 2015; Bedford et al., 2016). The older children also improved significantly on all measures across the 3 days, though not so markedly as the toddlers. They showed increased success and efficiency by 9.1 and 41.15%, respectively; and decreases in concept and motor errors, prompts, and completion times of 23.69, 42.19, 62.17, and 33.68%, respectively. Interestingly, apart from the motor skill measure, there were no interactions of age and trials. Both age groups improved at the same rate across trials and practice or experience did not differentially affect toddlers and the older children. The interpretation of the interaction with motor skills is unclear.

In contrast, the e-storybook task made less demand on children’s fine motor or navigational skill than did the shape matching task. Although they did have to search to find the features and tap to activate them and to move through the e-book, these are the earliest and simplest gestures to appear in toddlers’ repertoire (Aziz et al., 2014; Hourcade et al., 2015). Children had little difficulty with this as they explored and activated the features of interest about 28 times in the 7.39-min reading. The most important finding was that toddlers recognized more of the story content from the e-storybook than from the matched paper book. They were also more attentive (i.e., showed fewer distracted looks off task) during the e-book, though it took significantly longer to finish than did the paper book. This additional time might have been related to children’s engagement with the e-book features. It might also have provided children with more time to process the story details and enabled better content recognition. As there are few studies comparing toddlers’ learning from e-books and paper books, especially those with commercially available content, these results should be interpreted with caution pending replication (but see Strouse and Ganea, 2017a, b). Stories with a more traditional narrative structure, with more numerous or story irrelevant features, dialogic adult scaffolding, or those in which different or additional measures of retention are included (e.g., recall, story retelling or sequencing) might provide different results (Reich et al., 2016). Indeed, studies comparing book formats in older preschoolers have shown mixed results (Bus et al., 2015). Although a common finding is that they are more engaged in e-books, there is little evidence that they are consistently superior to traditional paper books for learning (Parish-Morris et al., 2013; Richter and Courage, 2017; O’Toole and Kannass, 2018). What the results reported here do indicate is that toddlers are very attentive to e-book stories and can engage with the app affordances as they listen to, and retain at least some of the story information.

Concerning the third research question, the results showed that children’s executive functions as assessed by the MEFS were a significant predictor of their success on the respective outcome measures of the shape matching and storybook apps. This is consistent with existing studies in which executive functioning has been shown to predict aspects of emergent literacy, math performance, spatial knowledge, and school achievement from measures in children as young as 2 years (Verdine et al., 2014b; De Franchis et al., 2017; Mulder et al., 2017; Purpura et al., 2017). In any learning context, children with stronger executive functions would predictably have more working memory capacity to attend to several streams of information at once, to focus and shift attention to the important elements of the task, and to inhibit distraction from extraneous sources (Blair and Razza, 2007; Fisch, 2017). As using touchscreen devices can be effortful (e.g., Zack et al., 2013; Fisch, 2017; Russo-Johnson et al., 2017), a child with stronger executive functions might have a performance advantage when using these devices to achieve a goal. Considering the shape matching app, children had to coordinate the motor skills required to navigate the pieces around the screen while keeping in mind the goal of finding the corresponding locations and then dropping the pieces. Similarly, using the interactive features of the e-book required children to switch attention away from the narration to search for and activate the feature with a tap and then reengage with the narration.

Finally, it is important to note that the children engaged in both of the app activities in the presence of an adult who guided their performance and kept their attention focused on the task. This scaffolding has been critical to toddlers’ successful learning from traditional, non-interactive screen media (see Barr, 2019) and is likely also the case for interactive devices (Samarakoon et al., 2019). Though not tested in the current study, Walter-Laager et al. (2016) found that 23- to 31-month-old toddlers who used a word-learning app scaffolded by an adult, had a larger vocabulary gain than those who used the app alone. Interestingly, some apps and e-books now contain built-in software features designed to simulate adult scaffolding (Bus et al., 2015). These include definitions, prompts, feedback, dialogic questions, pointing, and non-social contingent instructions (e.g., a “ghost” demonstration). Although some have been effective for older preschoolers (Takacs et al., 2014; Kwok et al., 2016; Strouse and Ganea, 2016), there is evidence that they are not as effective for toddlers as is having social contingency provided in person, especially if the problem is complex and likely to tax their cognitive resources (Moser et al., 2015; Zimmerman et al., 2017; Antrilli and Wang, 2018). A related concern is the broader issue of the impact of reduced or altered parent-child interactions when joint play or learning activities are in electronic format (Wooldridge and Shapka, 2012; Parish-Morris et al., 2013; Zosh et al., 2015; Verdine et al., 2016; Munzer et al., 2019). This will be a question for future research as more apps are designed for solitary play and learning (Takacs et al., 2014; Kucirkova and Zuckerman, 2017).

### Limitations to the Study

Although the results of the research cautiously support the appropriate use of well-designed apps for toddlers, there were several limitations to the study. First, the children comprised a convenience sample who happened to be from well-educated families in which most parents had at least one university degree. Whether the findings would generalize to children from a different demographic remains unclear. Second, the study took place in the children’s childcare centers where they participated in the activities with an unfamiliar adult. Although they were attentive and cooperative, they rarely engaged the adult in conversation. As parent-child conversation supports learning during traditional shared reading and shape skill activities at home, the more formal research context did not reflect a typical learning interaction. Third, the sole measure of retention for the storybook activity was a picture recognition test. Although a recognition score can be inflated, it may be a more sensitive reflection of toddlers’ retention than the more rigorous, standard measure of recall that would likely underestimate performance in children whose language production is immature. Finally, the story reading activity in both formats took place only once. Repeated opportunities such as children likely experience at home might have been more informative than a single trial.

## Conclusion

This study was among the first to show that children as young as 2-years of age were enthusiastic and attentive to an interactive touchscreen device and could learn to operate it purposefully to achieve a goal or to enhance a story. Importantly, the study also showed that those with more mature executive functions were particularly skilled using the apps as were a comparison group of 3-year-olds. The results add to a growing literature on the cognitive contents and skills (e.g., visuospatial, narrative, navigational) that toddlers can acquire from commercially available apps such as those they might have access to at home. This is important as the choice and availability of apps for toddlers that have educational content based on the science of learning (Hirsh-Pasek et al., 2015; Dore et al., 2019) will only increase. Some questions for future research include: (1) the logistics of app design that is age-appropriate and optimized for simple and intuitive use by toddlers as they explore the cognitive contents embedded in the material, (2) the nature of scaffolding that will best support and focus the user’s attention while fostering the learning independence that is inherent in a well-designed app, and (3) the transfer of skills and content learned in electronic formats to real-world examples. Finally, although apps will unlikely replace traditional shape skill toys or paper storybooks any time soon, the evidence to date suggests that they might prove to be a valuable addition to the toolbox of activities including, children’s spatial understanding and story comprehension more broadly. As such, they will continue to provide an alternative way to motivate, entertain, and instruct young children that will complement the traditional formats and could have implications for app design and policy development.

## Data Availability Statement

The raw data supporting the conclusions of this article will be made available by the authors, without undue reservation.

## Ethics Statement

The studies involving human participants were reviewed and approved by Institutional Committee on Ethics in Human Research. Written informed consent was obtained from the minor(s)’ legal guardian/next of kin for the publication of any potentially identifiable images or data included in this article.

## Author Contributions

MC designed and supervised the study and data analysis and wrote the manuscript. LF, CW, and MS assisted in planning the study, recruited and tested participants, and organized the data. All authors contributed to the article and approved the submitted version.

## Conflict of Interest

The authors declare that the research was conducted in the absence of any commercial or financial relationships that could be construed as a potential conflict of interest.

## Footnotes

1 tinyhandsapps.com
